# Preserving or peeling the inferior mesenteric arterial sheath during laparoscopic rectal cancer surgery: a prospective study of surgical outcomes

**DOI:** 10.1186/s12893-023-02083-7

**Published:** 2023-06-27

**Authors:** Qian Li, Ye Wang, Jia-wei Wang, Long Qian, Song Wang, Ting-ting Cao, Ya-bin Xia, Xiao-xu Huang, Li Xu

**Affiliations:** 1 Department of Gastrointestinal Surgery, First Affiliated Hospital of Wannan Medical College, Yijishan Hospital of Wannan Medical College, No. 2, Zheshan West Road, Wuhu, Anhui, 241001 China; 2 Key Laboratory of Non-coding RNA Transformation Research of Anhui Higher Education Institution, Wuhu, Anhui China; 3 Non-coding RNA Research Center of Wannan Medical College, Yijishan Hospital, Wuhu, Anhui China

**Keywords:** Rectal cancer, Laparoscopic surgery, Inferior mesenteric artery, Sheath, Lymph nodes, Prospective study

## Abstract

**Background:**

We mainly evaluated whether preserving the inferior mesenteric artery (IMA) sheath to dissecting IMA root lymph nodes (also called No.253 lymph nodes) would benefit patients in terms of comparable lymph-node yield removed during operation and postoperative complications in laparoscopic radical resection of rectal cancer.

**Methods:**

This is a prospective study included 141 rectal cancer patients who received laparoscopic radical resection during September 2018 to December 2020. All patients were randomly assigned to the preserved group (n = 71) and the peeled group (n = 70). The baseline characteristics, pathological features, intraoperative and postoperative data outcomes and complications were analyzed by independent samples *t* test, chi-square test or Fisher’s exact test between the 2 groups.

**Results:**

The baseline characteristic and pathological features had no statistical difference between the 2 groups. The preserved group had a shorter operative time (*P* = 0.002), a shorter lymph node dissection time (*P* < 0.001), less intraoperative bleeding (*P* = 0.004), an earlier time to first flatus (*P* = 0.013), an earlier time to fluid intake (*P* = 0.033) and a shorter length of hospitalization (*P* = 0.012) than the peeled group. The differences between the 2 groups were not statistically significant (*P* > 0.05) in regard to the total number of lymph nodes cleared, positive lymph nodes, bleeding, anastomotic leakage, pneumonia, wound infection, abscess, ileus, urinary retention, urinary tract infection and chyle leakage.

**Conclusion:**

Preserving of the IMA sheath in laparoscopic radical surgery for rectal cancer will reduce the total operation time and the length of hospitalization. This surgical method could lead to lower complication rate and faster recovery.

**Trial registration:**

The study was approved by the Ethics Committee of The First Affiliated Hospital of Wannan Medical College and registered by the China Clinical Trials Registry (ChiCTR2200060830, Date of Registration:2022-06-12 -retrospective registration) http://www.chictr.org.cn/index.aspx.

## Introduction

There were 4.064 million new cases of cancer and 2.413 million new deaths occurred in 2016 and showing a continuing upward trend in China [1]. The colorectal cancer is the second most common cause of cancer death, with the incidence in adults age 50 years or younger increasing by 1.5% per year from 2014 to 2018 in the USA, and the 5-year survival rate in the UK is only 50% [2–4]. Therefore, it is important for doctors to improve treatment outcomes in colorectal cancer patients.

Even so, there are numerous ways to treat rectal cancer, including surgery, radiation, chemotherapy, molecular targeted therapies, immunotherapies, and Chinese herbal medications. Some molecular targeted drugs, such as cetuximab, panitumumab and bevacizumab, have been shown to play important roles in the treatment of rectal cancer [5–7]. Some immunotherapy drugs that can help improve the immune system of rectal cancer patients, react and kill tumor cells, such as nivolumab and pembrolizumab, have been developed and have achieved good clinical efficacy, and some drugs have even been included in the treatment guidelines for colorectal cancer [8–10]. Like artemisinin, which can effectively treat malaria, Chinese herbal medicine has achieved therapeutic effects on colorectal cancer in previous practices. Some special ingredients contained in Chinese herbal medicine can kill tumor cells, and the treatment of colorectal cancer with traditional Chinese medicine has been included in the treatment guidelines of CSCO [11–13]. Laparoscopic radical resection of rectal cancer is a key component of these techniques. As it results in less surgical damage and accomplishes better lymph node dissection than traditional laparotomy, laparoscopic radical surgery for rectal cancer is becoming more and more popular among surgeons [14, 15].

Different scholars have different opinions regarding laparoscopic radical surgery for rectal cancer, and many studies have been performed on the dissection of No.253 lymph nodes, but few clinical studies have been conducted on the preservation of the IMA sheath during the dissection of No.253 lymph nodes. In the past, we thought that peeling the IMA sheath would increase the detection rate of No.253 lymph nodes in patients with rectal cancer [16]. But now scholars have the opposite opinion. Professor Gong ‘s [17] research findings that peeling the IMA sheath can increase surgical and anesthesia time, and may also cause bleeding damage to the IMA, thereby increasing the surgical risk for patients. Compared to preserving the vascular sheath, peeling the vascular sheath does not increase the number of lymph node dissection and improve the quality of surgery.

We designed this prospective randomized controlled study to investigate whether preserving the IMA sheath is more advantageous in lymph node dissection treatment and postoperative complications compared to peeling the IMA sheath to clean the lymph nodes at the root of the IMA.

## Materials and methods

### Study population

This is a prospective randomized controlled trial included 141 rectal cancer patients who received laparoscopic radical resection during September 2018 to December 2020, in the Department of Gastrointestinal Surgery, The First Affiliated Hospital of Wannan Medical College. All patients were prospectively and randomly assigned to the preserved group (n = 71) and the peeled group (n = 70) by envelope method. Both groups of patients underwent Dixon surgery for laparoscopic radical resection of rectal cancer, while the patient’s left colic artery (LCA) was preserved during the surgery. Patients with preserved IMA sheaths were placed in the preserved group, and patients with peeled IMA sheaths were placed in the peeled group. All patients and their families communicated with providers and signed informed consent forms before surgery. This study had been performed in accordance with the principles stated in the Declaration of Helsinki. The study was approved by the Ethics Committee of The First Affiliated Hospital of Wannan Medical College and registered by the China Clinical Trials Registry (ChiCTR2200060830).

### Inclusion and exclusion criteria

The inclusion criteria were as follows: preoperative pathology showing adenocarcinoma, preoperative diagnosis of stage T1-T3(according to the American Joint Committee on Cancer (AJCC) 7th edition TNM tumor staging criteria) rectal cancer by CT or MRI with a range of 5-15 cm from the anal verge, single rectal tumor lesion with no distant metastases, preoperative American Society of Anesthesiologists (ASA) rating of I-III, no emergency surgery, and no preoperative radiotherapy or other antineoplastic treatment.

**Fig. 1:**
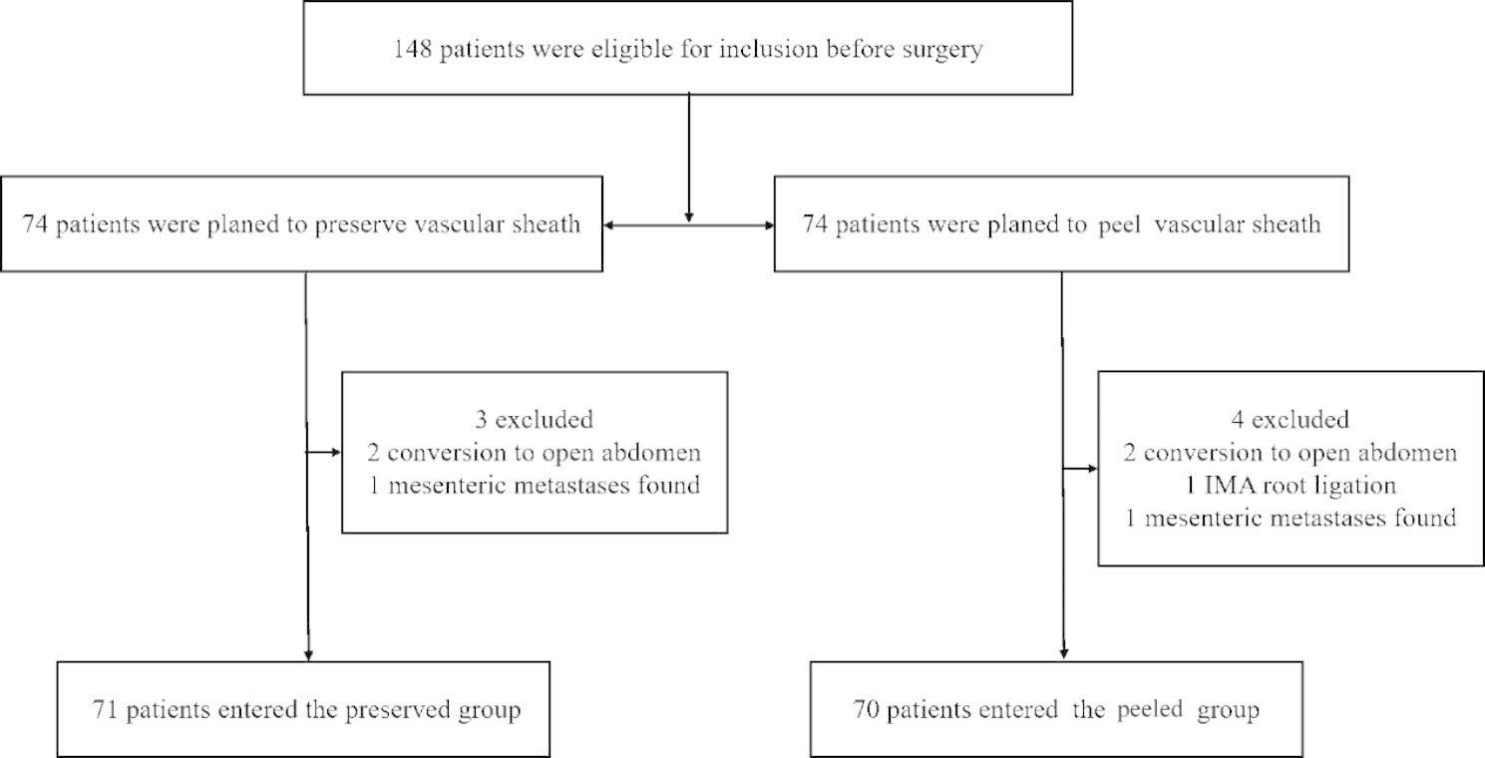
Flow chart of the study profile

Exclusion criteria were as follows: conversion to open surgery during laparoscopic surgery; intraoperative finding of mesenteric metastases or local invasion; intraoperative IMA root ligation for bleeding; and patients with comorbid systemic diseases that are inoperable, such as severe coagulation dysfunction, severe liver disease, severe renal disease, severe cardiac disease, severe pulmonary disease or other systemic malignancies. All patients received oral antibiotics 3 days prior to surgery to prevent infection and a cleansing enema 1 day prior to surgery. Those with one or more of the above conditions were excluded. A flow diagram for the study participant screening and grouping is shown in Fig. 1.

### Randomization

The researchers, not the operators, put one of the two surgical schemes into an opaque envelope which has the same appearance and size and 100 envelopes are allocated for each scheme. After these envelopes are completely messed up, write a code on the outside of the envelope, seal it and give it to the operator. When a research object enters the study, if it meets the inclusion criteria and exclusion criteria, number the patient, open the corresponding numbered envelope, and perform the surgery according to the grouping scheme in the envelope. The treatment plan received by each subject was determined by the generated random sequence.

### Surgical methodology

**Fig. 2:**
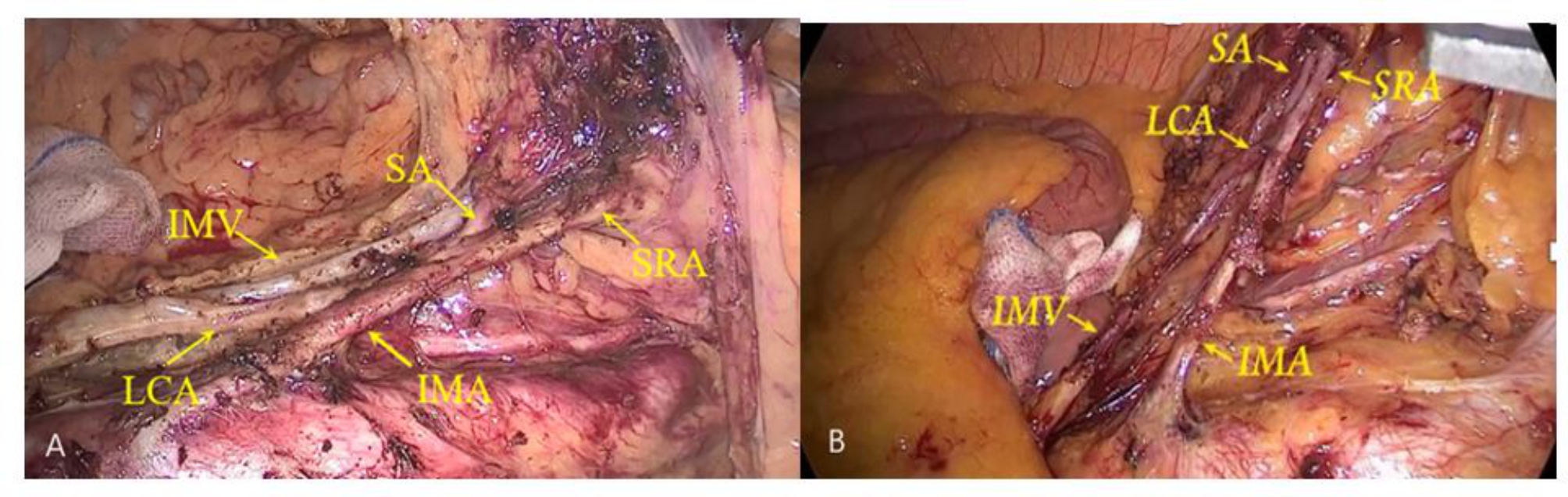
**(A)**: The No. 253 lymph nodes were dissected by preserving IMA sheath. **(B)**: The No. 253 lymph nodes were dissected by peeling IMA sheath

**Fig. 3:**
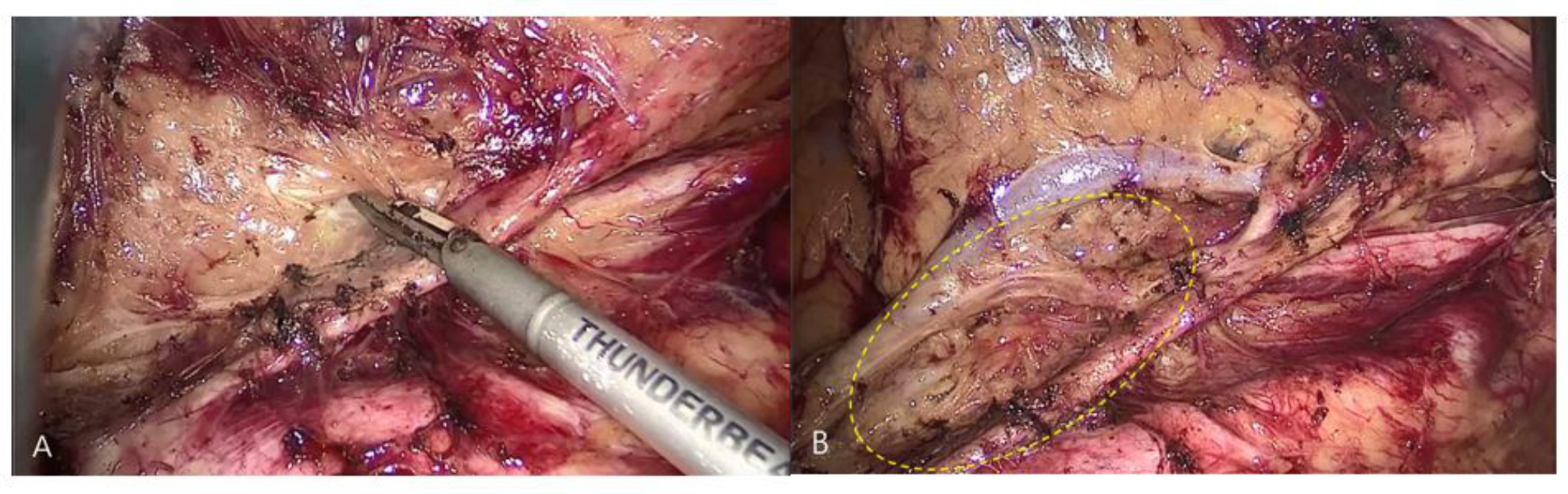
**(A)** and **(B)** show a before-and-after comparison of the dissection of No. 253 lymph nodes. The area of No. 253 lymph nodes dissection is indicated by the yellow dotted area in figure B

All the operations were performed by the same team of surgeons following the established grouping requirements. The patient was placed in a supine head-high, leg-low position under general anesthesia via tracheal intubation. Disinfecting the skin, and laying sterile sheets routinely, the abdominal cavity was routinely explored, peeling the right paramedian rectal sulcus with an ultrasound knife. The left mesorectal was separated along the abdominal aorta, the inferior mesenteric plexus was exposed, the IMA was exposed, and lymph node dissection from the beginning of the IMA to the beginning of the LCA without peeling the IMA sheath was performed in the preserved group. In the preserved group, the IMA sheath was not peeled, and the lymph nodes and adipose tissue in the No.253 lymph nodes area were dissected outside the IMA sheath. In the peeled group, the IMA sheath was peeled, and the lymph nodes were dissected at No.253 lymph nodes, the LCA, sigmoid artery (SA) and superior rectal artery (SRA) were exposed. To peel the IMA sheath, we gently peeled the IMA sheath from the root of the IMA with the ultrasound knife and then carefully and gently pushed the nonworking side of the ultrasound knife through the gap between the sheath and the vessel to the bifurcation of the IMA and the LCA, peeling away the intact IMA sheath. Before peeling the IMA sheath, we would carefully free the lymph and adipose tissue at the root of the IMA, expose and protect the root nerve plexus that runs along both sides of the IMA root. Generally, we operated from the point 0.5 cm away from the connection between the IMA and the abdominal aorta to avoid thermal damage to the nerves and blood vessels. The surrounding adipose tissue and lymph nodes were gently dissected, completing the dissection of the No.253 lymph nodes (Fig. 2). The No.253 lymph nodes were cleared, the inferior mesenteric vein (IMV) was ligated at the intersection with the LCA, and the SA and SRA were ligated and dissected. The area for No.253 lymph nodes dissection was delineated by the IMV on the left, the abdominal aorta on the right, the duodenum on the cephalic side and the area of the angle between the IMA and the LCA on the caudal side (Fig. 3). We dissected the sigmoid and descending colonic mesentery and loosened the lateral mesentery to the splenic area if tension was excessive. We exposed the posterior rectal space downward and the inferior epigastric plexus and separated the sides of the anterior sacral space to the pararectal sulcus, the lateral space, the anterior rectal space, and the lower edge to 2 cm below the tumor, taking care to protect the plexus and the bare rectal bowel wall. The distance to the lower margin of rectal resection was determined, the rectum was cut by a linear cutter stapler (Echelon Flex, Johnson & Johnson) at least 2 cm away from the lower edge of the tumor. Then we used a 3–5 cm vertical incision below the umbilicus, beyond this length we defined it as an intraoperative transverse abdomen, and the severed intestinal canal was pulled out from the abdominal cavity. Then, we trimmed the colon mesentery to better remove the tumor and perform intestinal anastomosis and cut the intestinal canal at a distance of 10 cm from the upper edge of the tumor. An iodine gauze strip was used to disinfect the intestinal cavity. A circular stapler anvil (Echelon Flex, Johnson & Johnson) was placed in the proximal intestinal canal, tied and fixed in the intestinal canal before putting it into the abdominal cavity. The assistant disinfected the external skin of the anus with iodine gauze again, relaxed and expanded the anus with fingers, and then placed a circular stapler (Echelon Flex, Johnson & Johnson) from the anus to anastomose with the anvil in the abdominal cavity. Injected about 50-100ml of diluted iodine solution from the anus into the intestinal canal to check if there were any gaps at the anastomosis. Then, reinforced and sutured the anterior wall of the rectum with absorbable suture at the anastomosis site.

### Statistical analysis

All categorical data were measured in numbers or percentages. Microsoft Excel was used for clinical data collection. Statistical Package for Social Science (version 23.0; IBM Corp) software was used for statistical analysis of the data. Values were expressed as ‾x ± s, and independent samples *t* test was used for comparison of both groups. Categorical data were compared using the chi-square test (χ2) or Fisher’s exact test. The difference was statistically significant at *P* < 0.05.

## Results

A total of 141 patients underwent laparoscopic radical rectal cancer treatment in our hospital between September 1, 2018, and December 31, 2020. They were randomly divided into the preservation group and the peeled group, and the general clinical data of the 2 groups were compared in Table 1. No significant differences were observed in the baseline characteristics of the groups (P > 0.05).

**Table 1:** Baseline characteristics

Characteristics	Preserved group (n = 71)	Peeled group (n = 70)	*p* Value
Age, year	62.8 ± 10.3	63.6 ± 9.2	0.611
Sex			0.810
Male	44(62.0)	42(60.0)	
Female	27(38.0)	28(40.0)	
Body mass index, kg/m2	22.8 ± 2.5	23.0 ± 2.7	0.264
Tumor diameter (cm)	3.2 ± 1.3	3.4 ± 1.6	0.231
Lymphatic/vascular invasion			0.806
Yes	16(22.5)	17(24.3)	
No	55(77.5)	53(75.7)	
Perineural invasion			0.804
Yes	15(21.1)	16(22.9)	
No	56(78.9)	54(77.1)	
Differentiation			0.811
Well to moderate	51(71.8)	49(70.0)	
Poor	20 (28.2)	21(30.0)	
ASA grade			0.154
I	14(19.7)	15(21.4)	
II	48(67.6)	47(67.1)	
III	9(12.7)	8(11.4)	
pTNM stage			0.923
T_1_	14(19.7)	12(17.1)	
T_2_	22(31.0)	22 (31.4)	
T_3_	35(49.3)	36(51.4)	

### Intraoperative data outcomes

Preserved group had a shorter No.253 lymph nodes dissection time (15.7 ± 4. 2 min vs. 26.0 ± 3. 6 min, *P* < 0.001), a shorter operative time (162.1 ± 20. 6 min vs. 174.4 ± 26. 0 min, *P* = 0.002), and less intraoperative bleeding (39.9 ± 7. 0 ml vs. 44.6 ± 11. 3 ml, *P* = 0.004) (Table 2) than the peeled group.

**Table 2:** Operative outcomes

Outcomes	Preserved group (n = 71)	Peeled group (n = 70)	*p* value
Operative time, min	162.1 ± 20.6	174.4 ± 26.0	0.002
No.253 lymph nodes dissection time, min	15.7 ± 4.2	26.0 ± 3.6	< 0.001
Intraoperative bleeding, ml	39.9 ± 7.0	44.6 ± 11.3	0.004

Operative time was defined as the time from incision to skin closure. No.253 lymph nodes dissection time was defined as the time between the start of lymph node dissection at No.253 lymph nodes and the end of regional dissection. Operative bleeding was obtained from the perioperative care record sheet.

### Pathological outcomes

There was no statistically significant difference between the 2 groups in terms of the number of lymph nodes dissected, the number of No.253 lymph nodes dissected, the number of positive No.253 lymph nodes and number of patients with No.253 lymph nodes involvement (P > 0.05, Table 3).

**Table 3:** Pathological outcomes

Outcomes	Preserved group (n = 71)	Peeled group (n = 70)	*p* value	
Total lymph nodes, n	19.3 ± 3.3	19.5 ± 3.1		0.765
No.253 lymph nodes, n	2.5 ± 1.1	2.8 ± 1.0		0.094
Positive No.253 lymph nodes, n	0.07 ± 2.6	0.07 ± 2.6		0.982
Patients with No.253 lymph nodes involvement, n	5.0	5.0		1.000

### Postoperative recovery and complications

The preserved group had an earlier time to first flatus (*P* = 0.013), earlier time to fluid intake (*P* = 0.033) and shorter hospitalization (*P* = 0.012). The differences between the 2 groups were not statistically significant (*P* > 0.05. Table 4) for anastomotic leakage, pneumonia, wound infection, abscess, deep vein thrombosis, ileus, urinary preserved, urinary tract infection and chyle leakage.

**Table 4:** Postoperative recovery and complications

Outcomes	Preserved group (n = 71)		Peeled group (n = 70)		*p* value	
Postoperative mortality	0		0		NE	
Postoperative bleeding	1 (1.4%)		2 (2.9%)		0.551	
Time to first flatus, days	2.4 ± 0.7		2.7 ± 0.8		0.013	
Time to fluid intake, days	3.2 ± 1.1		3.7 ± 1.3		0.033	
Hospitalization, days	9.1 ± 1.3		9.9 ± 2.0		0.012	
Anastomotic leakage, n	2 (2.8%)		4 (5.7%)		0.664	
Pneumonia, n	3 (4.2%)		3 (4.3%)		0.986	
Wound infection, n	3 (4.2%)		4 (5.7%)		0.985	
Abscess, n	1 (1.4%)		3 (4.3%)		0.602	
Deep vein thrombosis, n	0		0		NE	
Ileus, n	2 (2.8%)		3 (4.3%)		0.987	
Urinary retention, n	5 (7.0%)		6 (8.6%)		0.735	
Urinary tract infection, n	3 (4.2%)		5 (7.1%)		0.700	
Chyle leakage, n	1 (1.4%)		2 (2.9%)		0.551	

Postoperative bleeding was defined as bleeding requiring hemostatic medication or surgical intervention to stop it. Abscess was defined as a postoperative abscess that appeared around the anastomosis or elsewhere in the abdominal cavity. Chyle leakage was defined as a milky white drainage fluid with all positive qualitative tests for chyle and Sudan III staining tests.

## Discussion

We cleaned the lymph nodes at the root of the IMA during laparoscopic rectal cancer radical surgery by preserving the IMA sheath and peeling the IMA sheath. The purpose of designing this trial is to compare the outcomes of lymph node dissection and complications of postoperative between the two surgical methods to explore the advantages and disadvantages of the two surgical methods. The study results indicate that peeling the IMA sheath does not significantly increase the number of lymph node dissection in patients, and does not demonstrate a significant advantage in postoperative complications. However, it can increase the time for surgery and anesthesia in patients.

TNM staging (which involves the number of positive lymph nodes) plays an important role in the treatment strategy and prognosis of colorectal cancer, including in peel or laparoscopic surgery or even surgery assisted by the da Vinci robot. Before the appearance of distant metastases, lymph node metastasis is a critical prognostic factor for patients with colorectal cancer and is related to the subsequent treatment plan and course of treatment [18]. Chinese and Japanese scholars have emphasized the importance of lymph node dissection and the importance of dissecting lymph nodes that may have metastases intraoperatively, which has also received increasing attention from Western scholars [19]. In total mesorectal excision, the effectiveness of lymph node dissection is particularly important; therefore, as many lymph nodes as possible need to be dissected to prevent postoperative recurrence.

The lymph nodes at the root of the IMA are classified as third station lymph nodes of the sigmoid colon or rectum. Most scholars think that the area for No.253 lymph nodes dissection is delineated by the IMV on the left, the abdominal aorta on the right, the duodenum on the cephalic side and the area of the angle between the IMA and the LCA on the caudal side [20, 21]. No.253 lymph nodes are located between the beginning of the IMA and the beginning of the LCA. When the mesentery is opened at the level of sacral promontory, the IMA root lymph nodes can be clearly visualized in a high-definition laparoscopic view. Around the IMA root, there is a complex network of lymphatic vessels that run together, draining cephalad to the lumbar lymphatic pools on both sides along the abdominal aorta to the periaortic lymph nodes, and they receive lymphatic drainage from the sigmoid colon and rectum. From the pathway of rectal lymphatic drainage, low rectal cancer patients are at risk of No.253 lymph nodes metastasis; therefore, routine No.253 lymph nodes dissection is recommended for rectal cancer [22, 23].

The surgical approach of this trial was performed in accordance with the standard laparoscopic radical rectal cancer surgery (Dixon procedure) with preservation of the LCA, while the lymph nodes in No.253 lymph nodes were cleared according to the established requirements for the preserved or peeled groups, and the LCA was preserved because previous research has shown that preserving the LCA in radical rectal cancer surgery can increase the blood supply to the proximal IMA of the colon, provide better conditions for the growth and healing of the anastomosis, reduce the incidence of anastomotic leak and anastomotic stenosis postoperatively and is convenient during laparoscopy [24–26] .

**Fig. 4:**
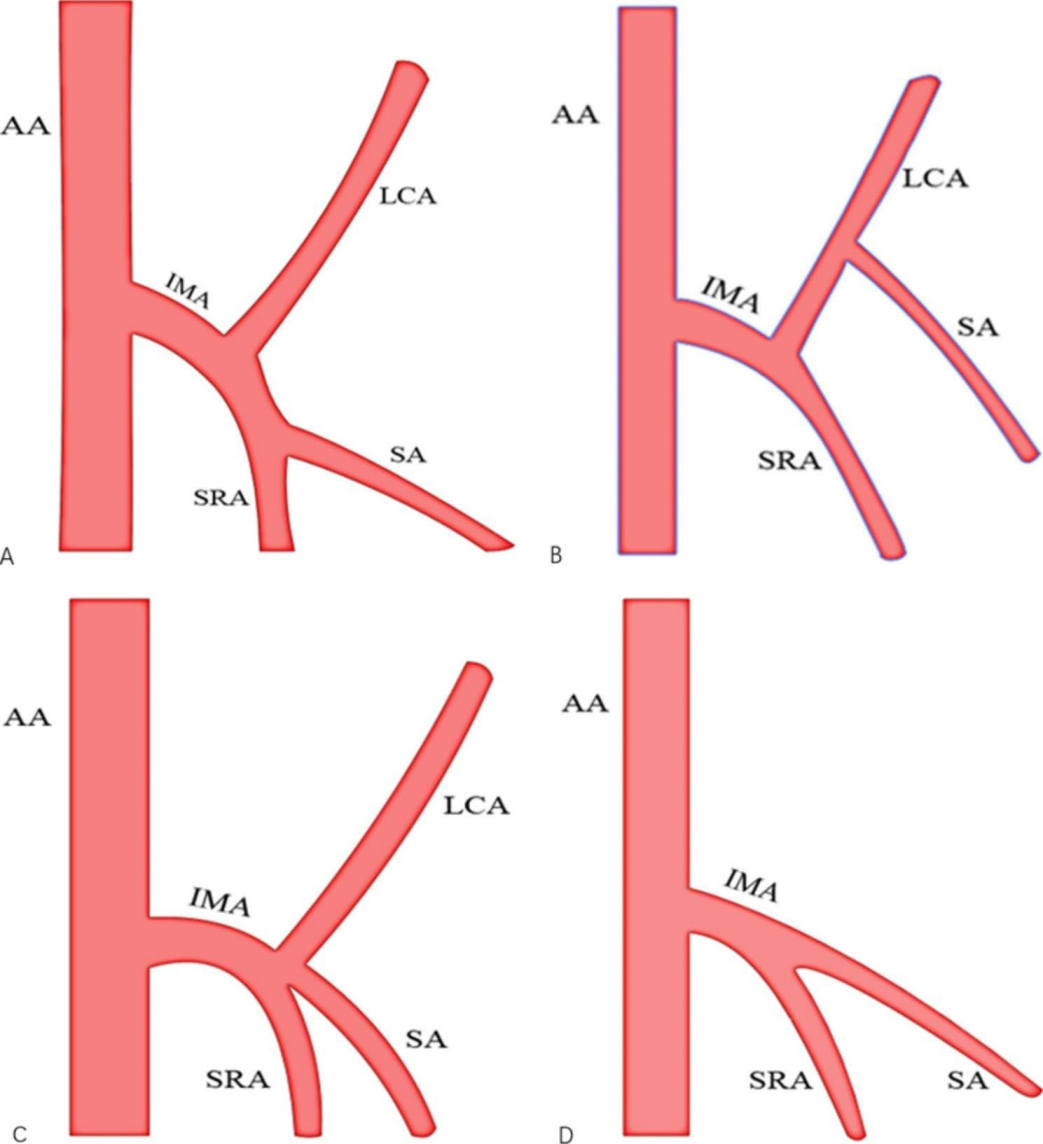
IMA subtypes: **(A)** Type I: the LCA branches first, followed by the SA and SRA ; **(B)** Type II: IMA first divides into one branch, which is the common branch of LCA and SA, SRA is a single branch, and then the common branch is divided into rising LCA and falling SA; **(C)** Type III: the LCA, SA and SRA branch from the IMA simultaneously at a node; **(D)** Type IV: the LCA is absent ,and the IMA divides directly into the SA and SRA

The influence of heteromorphosis of IMA vascular branches on the surgical approach to peeling the sheath should be noted when dissecting No.253 lymph nodes. For IMA vascular typing, Yada [27] and Shen [28] classified IMA into four types (type I to type IV, Fig. 4) based on the relationship between the LCA, SA and the root of the SRA as follows: in type I ,the LCA branches first, followed by the SA and SRA ; in type II: IMA first divides into one branch, which is the common branch of LCA and SA, SRA is a single branch, and then the common branch is divided into rising LCA and falling SA; in type III, the LCA, SA and SRA branch from the IMA simultaneously at a node; in type IV ,the LCA is absent ,and the IMA divides directly into the SA and SRA. Ke [29] performed a study measuring vascular branches of IMA statistics on 471 patients through vessel reconstruction and found that most patients had type III (n = 209, 44.7%), followed by type I patients (n = 193, 41.2%) and type II patients (n = 42, 9.0%), while type IV patients were the least numerous (n = 24, 5.1%). By understanding the IMA type, clinical surgeons can facilitate the intraoperative search, determination and precise preservation of the LCA and advance the design of the ligature and location of the SA and SRA vessels. Particularly, in patients with LCA deficiency. It is possible to avoid spending more operative time searching for nonexistent LCA in the naked IMA trunk, thus shortening the operative time, reducing the risk of unnecessary injuries and improving the efficiency and safety of the operation.

The IMA sheath is the tissue that lies between the outer surface of the IMA and the collagen layer that connects the outermost nerve fibers. It contains collagen fibers, nerves, microvasculature, lymphatic vessels and adipose tissue. Studies have shown that microscopically, the IMA sheath contains only lymphatic vessels without the presence of lymph nodes, and no tumor cells are observed within the IMA sheath in lymph node metastasis-positive cases [30, 31]. However, some oriental scholars believe that better lymph node dissection and ensuring the integrity of the resected mesentery are important means to avoid tumor cell omission during surgery. Therefore, in laparoscopic rectal cancer surgery, in order to avoid the tumor cells moving away along the lymphatic vessels in the sheath, and the root of IMA is the key metastasis path for middle and upper rectal cancer, So the dissection of IMA vascular sheath can treat tumor metastasis in this area or prevent the metastasis of rectal cancer to the cephalic side by cutting off the metastasis pathway here [22, 32, 33]. Following the principle of radical tumor resection and choroidal debridement, it is important to debride the extrathecal lymph nodes and intrathecal lymph nodes of the residual sheath as completely as possible.

On the basis of dissection of the IMA sheath. There are currently two types of lymph node dissection for No.253 lymph nodes, and one is to preserve the IMA sheath surgical approach. Li [34] and Gong [17] concluded that depending on the anatomy of the sheath, since there are only lymphatic structures and no lymph nodes in the sheath, stripping the sheath cannot change the lymph node staging of postoperative pathological tissue, nor can it affect the subsequent treatment. Meanwhile, the reported literature mostly comprises retrospective studies on the surgical approach of peeling the arterial sheath for lymph node dissection. Because of the lack of multicenter and large sample size prospective studies, the significance of peeling the sheath for lymph node dissection is unclear and not clearly recommended by relevant guidelines. Additionally, peeling the IMA sheath to dissect No.253 lymph nodes is clearly more difficult in patients who are elderly, obese, have arteriosclerosis or a history of abdominal surgery, and is likely to cause damage to arteries and nerves. The other approach is to operate without preserving the IMA sheath, such as Yan [22] and He [35], who think that the No.253 lymph nodes and the lymphatic vessels within the arterial sheath should be removed together from the consideration of the principle of radical resection in oncological surgery. At the same time, lymph nodes dissection at No. 253 lymph nodes was performed within the sheath, which could serve as a prophylactic dissection for patients without metastases in the sheath and could achieve radical treatment for those with metastases.

In this study, the preserved group had a shorter total operative time than the peeled group. The preservation of the IMA sheath for dissection reduces the operative time, makes the procedure simpler, and reduces the risk of intraoperative anesthetic accidents in patients with a long smoking history, especially those with comorbidities of diabetes, heart disease, kidney or liver problems, which helps to reduce operative risk, improve perioperative safety and improve patient prognosis [36]. The preserved group had less intraoperative bleeding than the peeled group. We can see that the specific difference of bleeding volume is not very large, which may be related to surgical proficiency or the use of ultrasonic scalpel reduces the intraoperative bleeding volume, but there is still a statistical significance between the two groups. We know that less bleeding can better achieve a clear intraoperative visual field, which helps to improve the safety of surgery and reduce the risk of intraoperative side injuries.

The preserved group had significantly shorter times to first flatus and fluid intake than the peeled group. Due to the presence of numerous capillaries and lymphatic vessels within the vascular sheath [37, 38], we think that preservation of the IMA sheath could well preserve the local blood and lymphatic return to the intestine and promote early recovery of intestinal function. Postoperative pathology showed no statistically significant difference in the total number of lymph nodes dissected, the number of No.253 lymph nodes cleared and the positive nodes between the 2 groups, indicating that peeling the IMA sheath to dissect No.253 lymph nodes did not increase the number of all lymph nodes cleared or the positive rate of lymph nodes. Moreover, it is possible that the length of hospitalization was also shorter in the preserved group because patients in the preserved group had shorter times to postoperative flatus and fluid intake.

There were no cases of postoperative mortality or deep vein thrombosis after the operation. Although there were cases of postoperative bleeding in the preserved and peeled group, they were all controlled by appropriate treatment. There were four anastomotic leakage patients in the peeled group (4/70, 5.7%) and only two in the preserved group (2/71, 2.8%). This might be due to the peeling of the sheath affecting the blood flow to the anastomosis, while the influence of the physical condition of the individual patients could not be excluded. Two of the patients with anastomotic leakage were older (both older than 70 years) or had underlying diseases such as hypertension and diabetes. Both groups had three patients with postoperative pneumonia, and all six patients had a long history of smoking. Data from the 2 groups showed no significant difference in the incidences of wound infection and abscess. The number of patients with postoperative ileus is lower than that in the past because surgeons pay more and more attention to the application of enhanced recovery after surgery (ERAS) concept in clinical work. Only two and three patients in both groups developed postoperative ileus; however, the symptoms were quickly alleviated with appropriate and timely treatment. Urinary retention infrequently occurred in both groups, and the data were not significantly different between the 2 groups (5/71, 7.0% vs. 6/70, 8.6% *P* = 0.735). There was no significant difference in terms of urinary tract infection between the 2 groups. With the use of an ultrasound knife in laparoscopic surgery, the number of patients with chyle leakage in laparoscopic rectal cancer surgery decreased significantly, but there were still cases of celiac disease in the reserved and peeled groups (1/71, 1.4% vs. 2/70, 2.9% *P* = 0.551). However, in this study, there was no statistically significant difference between the two datasets.

Although this study shows some significant results, there are still some limitations. First, this study is an independent single-center prospective randomized controlled trial, and further validation of the results is needed in a multicenter prospective study with a large sample size. Second, due to limitations in inclusion or exclusion criteria, further data were not available for some patients who had undergone preoperative radiotherapy or emergency cares, and a separate clinical trial could be designed in the future to verify the efficacy of surgery in this population. Finally, in order to reduce the impact of different surgical methods on No.253 of lymph node dissection, the surgical method adopted in this trial was to preserve the LCA for low anterior resection of rectal cancer. Whether the data brought by other surgical methods are similar to this trial needs further prospective trial to verify.

## Conclusion

Preserving of the IMA sheath in laparoscopic radical surgery for rectal cancer will reduce the total operation time and the length of hospitalization. This surgical method could lead to lower complication rate and faster recovery.

## Data Availability

The datasets generated and/or analysed during the current study are not publicly available but are available from the corresponding author on reasonable request.

## Abbreviations

IMA: Inferior mesenteric artery
AJCC: American Joint Committee on Cancer
ASA: American Society of Anesthesiologists
LCA: Left colic artery
SA: Sigmoid artery
SRA: Superior rectal artery
IMV: Inferior mesenteric vein
ERAS: Enhanced recovery after surgery
