# Correction to: Preserving or peeling the inferior mesenteric arterial sheath during laparoscopic rectal cancer surgery: a prospective study of surgical outcomes

**DOI:** 10.1186/s12893-023-02142-z

**Published:** 2023-08-18

**Authors:** Qian Li, Ye Wang, Jia-wei Wang, Long Qian, Song Wang, Ting-ting Cao, Ya-bin Xia, Xiao-xu Huang, Li Xu

**Affiliations:** 1 Department of Gastrointestinal Surgery, First Affiliated Hospital of Wannan Medical College, Yijishan Hospital of Wannan Medical College, No. 2, Zheshan West Road, Wuhu, Anhui, 241001 China; 2 Key Laboratory of Non-coding RNA Transformation Research of Anhui Higher Education Institution, Wuhu, Anhui, China; 3 Non-coding RNA Research Center of Wannan Medical College, Yijishan Hospital, Wuhu, Anhui, China

**Correction to: BMC Surgery**

10.1186/s12893-023-02083-7

Following publication of the original article [1], in this article the alignment for Tables 2 and 4 has been made and the same has been shown below:

**Table 2:** Operative outcomes

Outcomes	Preserved group (n = 71)	Peeled group (n = 70)	*p* value
Operative time, min	162.1 ± 20.6	174.4 ± 26.0	0.002
No.253 Lymph nodes dissection time, min	15.7 ± 4.2	26.0 ± 3.6	< 0.001
Intraoperative bleeding, ml	39.9 ± 7.0	44.6 ± 11.3	0.004

Operative time was defined as the time from incision to skin closure. No.253 lymph nodes dissection time was defined as the time between the start of lymph node dissection at No.253 lymph nodes and the end of regional dissection. Operative bleeding was obtained from the perioperative care record sheet

**Table 4:** Postoperative recovery and complications

Outcomes	Preserved group (n = 71)	Peeled group (n = 70)	*p* value
Postoperative mortality	0	0	NE
Postoperative bleeding	1 (1.4%)	2 (2.9%)	0.551
Time to first flatus, days	2.4 ± 0.7	2.7 ± 0.8	0.013
Time to fluid intake, days	3.2 ± 1.1	3.7 ± 1.3	0.033
Hospitalization, days	9.1 ± 1.3	9.9 ± 2.0	0.012
Anastomotic leakage, n	2 (2.8%)	4 (5.7%)	0.664
Pneumonia, n	3 (4.2%)	3 (4.3%)	0.986
Wound infection, n	3 (4.2%)	4 (5.7%)	0.985
Abscess, n	1 (1.4%)	3 (4.3%)	0.602
Deep vein thrombosis, n	0	0	NE
Ileus, n	2 (2.8%)	3 (4.3%)	0.987
Urinary retention, n	5 (7.0%)	6 (8.6%)	0.735
Urinary tract infection, n	3 (4.2%)	5 (7.1%)	0.700
Chyle leakage, n	1 (1.4%)	2 (2.9%)	0.551

Postoperative bleeding was defined as bleeding requiring hemostatic medication or surgical intervention to stop it. Abscess was defined as a postoperative abscess that appeared around the anastomosis or elsewhere in the abdominal cavity. Chyle leakage was defined as a milky white drainage fluid with all positive qualitative tests for chyle and Sudan III staining tests

The original article has been corrected.

